# Supplementary material for: Use of machine learning methods to understand discussions of female genital mutilation/cutting on social media
Source: PLOS Glob Public Health. 2023 Jul 25;3(7):e0000878. doi: 10.1371/journal.pgph.0000878 (PMC10368253; doi:10.1371/journal.pgph.0000878)
Supplement: S1 Table — Words are ordered based on their prevalence in 2015, followed by subsequent years. (DOCX) [file pgph.0000878.s001.docx]

S1 Table. Most Common Words Found in User Descriptions and The Proportion of User Descriptions Containing Each Word by Year

| Word | Proportion of User Descriptions Containing Word | | | | | |
| --- | --- | --- | --- | --- | --- | --- |
|  | 2015 | 2016 | 2017 | 2018 | 2019 | 2020 |
| news | 0.030 | 0.019 | 0.029 | 0.032 | 0.036 | 0.034 |
| views | 0.028 | 0.018 | 0.031 | 0.033 | 0.038 | 0.035 |
| health | 0.018 | 0.012 | 0.017 | 0.018 | 0.020 | 0.019 |
| love | 0.016 | 0.010 | 0.015 | 0.016 | 0.018 | 0.017 |
| rights | 0.015 | 0.010 | 0.013 | 0.015 | 0.017 | 0.016 |
| social | 0.014 | 0.009 | 0.013 | 0.014 | 0.016 | 0.015 |
| world | 0.013 | 0.008 | 0.011 | 0.013 | 0.013 | 0.013 |
| writer | 0.013 | 0.008 | 0.011 | 0.012 | 0.014 | 0.013 |
| life | 0.012 | 0.008 | 0.012 | 0.012 | 0.014 | 0.013 |
| trump | 0.009 | 0.007 | 0.011 | 0.012 | 0.014 | 0.012 |
| maga | 0.005 | 0.003 | 0.005 | 0.006 | 0.007 | 0.006 |
| music | 0.004 | 0.003 | 0.004 | 0.004 | 0.005 | 0.005 |
| conservative | 0.002 | 0.001 | 0.002 | 0.002 | 0.003 | 0.003 |
| god | 0.002 | 0.001 | 0.002 | 0.003 | 0.003 | 0.004 |
| politics | 0.002 | 0.002 | 0.002 | 0.002 | 0.003 | 0.003 |
| feminist | 0.002 | 0.001 | 0.002 | 0.002 | 0.002 | 0.002 |
| media | 0.002 | 0.001 | 0.002 | 0.002 | 0.002 | 0.002 |
| people | 0.002 | 0.001 | 0.002 | 0.002 | 0.002 | 0.002 |
| uk | 0.002 | 0.001 | 0.002 | 0.002 | 0.002 | 0.002 |
| human | 0.002 | 0.001 | 0.002 | 0.002 | 0.002 | 0.002 |
| tweets | 0.002 | 0.001 | 0.001 | 0.001 | 0.002 | 0.001 |
| fan | 0.002 | 0.001 | 0.001 | 0.001 | 0.002 | 0.002 |
| lover | 0.002 | 0.001 | 0.001 | 0.001 | 0.001 | 0.001 |
| advocate | 0.002 | 0.001 | 0.001 | 0.001 | 0.002 | 0.002 |
| proud | 0.001 | 0.001 | 0.001 | 0.002 | 0.002 | 0.002 |

Words are ordered based on their prevalence in 2015, followed by subsequent years.
