# Supplementary material for: Use of machine learning methods to understand discussions of female genital mutilation/cutting on social media
Source: PLOS Glob Public Health. 2023 Jul 25;3(7):e0000878. doi: 10.1371/journal.pgph.0000878 (PMC10368253; doi:10.1371/journal.pgph.0000878)
Supplement: S2 Table — Words are ordered based on their prevalence in 2015, followed by subsequent years. (DOCX) [file pgph.0000878.s002.docx]

S2 Table. Most Common Words Found in Tweet Text and The Proportion of Tweets Containing Each Word by Year

| Word | Proportion of Tweets Containing Word | | | | | |
| --- | --- | --- | --- | --- | --- | --- |
|  | 2015 | 2016 | 2017 | 2018 | 2019 | 2020 |
| endfgm | 0.219 | 0.237 | 0.236 | 0.250 | 0.256 | 0.264 |
| uk | 0.214 | 0.236 | 0.219 | 0.229 | 0.224 | 0.228 |
| circumcision | 0.159 | 0.161 | 0.154 | 0.148 | 0.145 | 0.142 |
| nigeria | 0.157 | 0.168 | 0.153 | 0.161 | 0.155 | 0.153 |
| end | 0.149 | 0.154 | 0.157 | 0.155 | 0.154 | 0.156 |
| child | 0.147 | 0.145 | 0.143 | 0.159 | 0.143 | 0.134 |
| egypt | 0.145 | 0.148 | 0.137 | 0.136 | 0.151 | 0.161 |
| news | 0.140 | 0.162 | 0.153 | 0.136 | 0.130 | 0.134 |
| doctor | 0.134 | 0.172 | 0.149 | 0.138 | 0.132 | 0.135 |
| today | 0.036 | 0.062 | 0.048 | 0.057 | 0.063 | 0.080 |
| kenya | 0.030 | 0.017 | 0.012 | 0.014 | 0.013 | 0.015 |
| stop | 0.016 | 0.012 | 0.013 | 0.014 | 0.014 | 0.017 |
| practice | 0.015 | 0.022 | 0.024 | 0.039 | 0.032 | 0.029 |
| banned | 0.011 | 0.008 | 0.009 | 0.010 | 0.013 | 0.015 |
| cutting | 0.010 | 0.008 | 0.011 | 0.010 | 0.012 | 0.013 |
| marriage | 0.010 | 0.011 | 0.013 | 0.020 | 0.017 | 0.012 |
| male | 0.010 | 0.014 | 0.011 | 0.020 | 0.017 | 0.018 |
| retweet | 0.008 | 0.011 | 0.015 | 0.018 | 0.040 | 0.046 |
| law | 0.008 | 0.005 | 0.007 | 0.007 | 0.008 | 0.008 |
| day | 0.008 | 0.008 | 0.011 | 0.014 | 0.021 | 0.021 |
| men | 0.007 | 0.039 | 0.016 | 0.014 | 0.014 | 0.018 |
| michigan | 0.007 | 0.007 | 0.006 | 0.008 | 0.013 | 0.009 |
| million | 0.006 | 0.005 | 0.007 | 0.026 | 0.014 | 0.014 |
| young | 0.006 | 0.003 | 0.004 | 0.004 | 0.006 | 0.007 |
| health | 0.006 | 0.010 | 0.025 | 0.011 | 0.012 | 0.011 |
| islam | 0.006 | 0.005 | 0.007 | 0.007 | 0.010 | 0.012 |
| sharia | 0.005 | 0.006 | 0.006 | 0.006 | 0.007 | 0.011 |
| muslim | 0.005 | 0.005 | 0.006 | 0.011 | 0.012 | 0.010 |
| rights | 0.005 | 0.004 | 0.005 | 0.005 | 0.004 | 0.012 |
| support | 0.004 | 0.003 | 0.005 | 0.005 | 0.006 | 0.006 |
| risk | 0.004 | 0.003 | 0.005 | 0.006 | 0.005 | 0.008 |
| people | 0.003 | 0.004 | 0.004 | 0.006 | 0.006 | 0.006 |
| gender | 0.003 | 0.003 | 0.004 | 0.004 | 0.006 | 0.006 |
| violence | 0.0028 | 0.003 | 0.004 | 0.005 | 0.005 | 0.005 |

Words are ordered based on their prevalence in 2015, followed by subsequent years.
