# Supplementary material for: Use of machine learning methods to understand discussions of female genital mutilation/cutting on social media
Source: PLOS Glob Public Health. 2023 Jul 25;3(7):e0000878. doi: 10.1371/journal.pgph.0000878 (PMC10368253; doi:10.1371/journal.pgph.0000878)
Supplement: S3 Table — The importance is ranked ordinally. The factor listed as “1” is the first most influential factor in engagement, the factor listed as “2” is the second most important, and so on. (DOCX) [file pgph.0000878.s003.docx]

S3 Table. Ranked Importance of Tweet and User Characteristics in Predicting High Retweets in Random Forest Model

| **Variables** | **2015**  (n=236,092)  90th ptile:  4 tweets | **2016**  (n=133,753)  90th ptile:  6 tweets | **2017**  (n=291,187)  90th ptile:  5 tweets | **2018**  (n=245,471)  90th ptile:  7 tweets | **2019**  (n=215,866)  90th ptile:  7 tweets | **2020**  (n=180,779)  90th ptile:  8 tweets |
| --- | --- | --- | --- | --- | --- | --- |
| Length of Tweet | 2 | 1 | 2 | 1 | 1 | 1 |
| Number of Followers | 1 | 2 | 1 | 2 | 2 | 2 |
| Number of Following | 3 | 3 | 3 | 4 | 3 | 3 |
| Number of Tweets | 4 | 4 | 4 | 3 | 4 | 4 |
| User Verification | 5 | 5 | 5 | 5 | 5 | 5 |
| **F1 Score** | 0.8150 | 0.8248 | 0.8242 | 0.8727 | 0.8924 | 0.8824 |

The importance is ranked ordinally. The factor listed as “1” is the first most influential factor in engagement, the factor listed as “2” is the second most important, and so on.
