# Supplementary material for: Use of machine learning methods to understand discussions of female genital mutilation/cutting on social media
Source: PLOS Glob Public Health. 2023 Jul 25;3(7):e0000878. doi: 10.1371/journal.pgph.0000878 (PMC10368253; doi:10.1371/journal.pgph.0000878)
Supplement: Attachment — Submitted filename: Response to Reviewers_2.docx [file pgph.0000878.s004.docx]

Dear Dr. del Mar Pastor Bravo,	March 1, 2023

Thank you for giving our team the opportunity to submit a revised draft of our manuscript titled “Use of machine learning methods to understand discussions of female genital mutilation/cutting on social media” to *PLOS Global Public Health*. We appreciate the time and effort that you and the reviewers have dedicated to providing your feedback on our manuscript. Changes are reflected in the included manuscript file. Responses to the editors and reviewers’ comments and concerns are below:

**Comments from the editors:**

*In your Methods section, please include additional information about your dataset and ensure that you have included a statement specifying whether the collection and analysis method complied with the terms and conditions for the source of the data.*

- Thank you for your comment. We’ve added an additional subsection on ‘Data extraction’ to ‘Materials and methods’ section: “We extracted tweets via Twitter’s Application Programming Interface (API), which provides a random 1% sample of all public tweets containing specific keywords. Tweets in our corpus contained at least one of the following terms: “FGM,” “female genital mutilation,” “female genitalia mutilation,” “female circumcision,” “female genital alteration,” “female genitalia alteration,” “female genital cutting,” “female genitalia cutting,” “female genital excision,” and “female genitalia excision.” The research was conducted in line with Twitter API’s requirements for academic research [22]. We restricted our tweets to tweets in English.”

*In the online submission form, you indicated that your data will be submitted to a repository upon acceptance. We strongly recommend all authors deposit their data before acceptance, as the process can be lengthy and hold up publication timelines. Please note that, though access restrictions are acceptable now, your entire data will need to be made freely accessible if your manuscript is accepted for publication. This policy applies to all data except where public deposition would breach compliance with the protocol approved by your research ethics board. If you are unable to adhere to our open data policy, please kindly revise your statement to explain your reasoning and we will seek the editor's input on an exemption. Please be assured that, once you have provided your new statement, the assessment of your exemption will not hold up the peer review process.*

- Thank you for bringing this to our attention. The data has been submitted to a repository that you can access here (https://github.com/graybabbs/fgmctwitter). We provide datasets containing tweet IDs for each of the six years in the dataset. Original tweet text and user metadata reported in this study cannot be deposited in a public repository because of Twitter's data sharing policy. Tweet IDs can be used to retrieve original tweet data via Twitter’s public application programming interface.

*Some material included in your submission may be copyrighted. According to PLOS’s copyright policy, authors who use figures or other material (e.g., graphics, clipart, maps) from another author or copyright holder must demonstrate or obtain permission to publish this material under the Creative Commons Attribution 4.0 International (CC BY 4.0) License used by PLOS journals. Please closely review the details of PLOS’s copyright requirements here: PLOS Licenses and Copyright. If you need to request permissions from a copyright holder, you may use PLOS's Copyright Content Permission form.*

*Please respond directly to this email or email the journal office and provide any known details concerning your material's license terms and permissions required for reuse, even if you have not yet obtained copyright permissions or are unsure of your material's copyright compatibility.*

*Potential Copyright Issues:*

*Figure 2: please (a) provide a direct link to the base layer of the map (i.e., the country or region border shape) and ensure this is also included in the figure legend; and (b) provide a link to the terms of use / license information for the base layer image or shapefile. We cannot publish proprietary or copyrighted maps (e.g. Google Maps, Mapquest) and the terms of use for your map base layer must be compatible with our CC-BY 4.0 license.*

*Note: if you created the map in a software program like R or ArcGIS, please locate and indicate the source of the basemap shapefile onto which data has been plotted.*

*If your map was obtained from a copyrighted source please amend the figure so that the base map used is from an openly available source. Alternatively, please provide explicit written permission from the copyright holder granting you the right to publish the material under our CC-BY 4.0 license.*

*Please note that the following CC BY licenses are compatible with PLOS license: CC BY 4.0, CC BY 2.0 and CC BY 3.0, meanwhile such licenses as CC BY-ND 3.0 and others are not compatible due to additional restrictions.*

*If you are unsure whether you can use a map or not, please do reach out and we will be able to help you. The following websites are good examples of where you can source open access or public domain maps:*

** U.S. Geological Survey (USGS) - All maps are in the public domain. (*[*http://www.usgs.gov*](http://www.usgs.gov/)*)*

** PlaniGlobe - All maps are published under a Creative Commons license so please cite “PlaniGlobe,* [*http://www.planiglobe.com*](http://www.planiglobe.com/)*, CC BY 2.0” in the image credit after the caption. (*[*http://www.planiglobe.com/?lang=enl*](http://www.planiglobe.com/?lang=enl)*)*

** Natural Earth - All maps are public domain. (*[*http://www.naturalearthdata.com/about/terms-of-use/*](http://www.naturalearthdata.com/about/terms-of-use)*)*

- Thank you for your comment and close attention to copyright issues. The base layer of Figure 2 was obtained via the ‘world’ dataset included in R’s maps package. This world map is imported from the public domain Natural Earth project. Information about the package may be found here: <https://cran.r-project.org/package=maps>. The two references for the ‘world’ dataset used are as follows:
  - Richard A. Becker, and Allan R. Wilks, "Maps in S", AT\&T Bell Laboratories Statistics Research Report [93.2], 1993.
  - Richard A. Becker, and Allan R. Wilks, "Constructing a Geographical Database", AT\&T Bell Laboratories Statistics Research Report [95.2], 1995.
- The license information for the R package may be found here: <https://cran.r-project.org/web/licenses/GPL-2>. The figure legend has been amended to include the Natural Earth map data citation as recommended in the Natural Earth terms of use.
- The terms of use for Natural Earth data may be found here: <https://www.naturalearthdata.com/about/terms-of-use/>.

**Comments from Reviewer 1:**

*This is the first time I reviewed this manuscript. I thought it was excellent. It's unique and well written. They are clear about their obvious limitations. I am not familiar with these methods but they appear sounds.*

- Thank you for your interest and kind words about the manuscript.

**Comments from Reviewer 2:**

*It reads more like a demonstration of a technique rather than a substantial contribution to the FGM debate. It is a neat demonstration, but ' so what'.* *On p. 5, 2nd paragraph the authors state what they will look at but no argument is made why exactly these aspects of the Twitter conversations are studied or what their relevance is.*

- Thank you for this feedback. We have edited this paragraph to clarify why we focused on the aspects of the FGM/C Twitter conversation that we did. Here is the amended paragraph: “In this paper, we use social media data to assess public perception of FGM/C on Twitter between 2015 and 2020. First, we characterize general discussions of FGM/C on English-speaking Twitter. Next, we analyze trends in discussion of FGM/C discussions on Twitter over the study period. Lastly, we characterize the meta-data of the discussion- what terms are people using to describe FGM/C, who is engaging in the conversation, and what influences engagement. Our findings can add to the current literature about public perception about FGM/C. Further, our work can be used by medical professionals, public health practitioners, governmental agencies, and advocacy organizations seeking to educate the public about FGM/C.”

*A demonstration of a new technique can be valuable but then the authors need to provide more details about how they went about it and what choices were made for what reasons. This is all lacking in this paper where the techniques and choices are only described in very general terms. Such demonstrations are only useful when they ' teach' how to use the technique. For instance, I would like to know how the tweets studied were selected. In meta-studies, for instance, one needs to specify in detail how the studies were selected. A similar approach would be useful here.*

- Thank you for your comment. We’ve added an additional subsection on ‘Data extraction’ to ‘Materials and methods’ section: “We extracted tweets via Twitter’s Application Programming Interface (API), which provides a random 1% sample of all public tweets containing specific keywords. Tweets in our corpus contained at least one of the following terms: “FGM,” female genital mutilation,” “female genitalia mutilation,” “female circumcision,” “female genital alteration,” “female genitalia alteration,” “female genital cutting,” “female genitalia cutting,” “female genital excision,” and “female genitalia excision.” The research was conducted in line with Twitter API’s requirements for academic research [22]. We restricted our tweets to tweets in English. ”

*In figure 1 the authors list the most frequent terms in 2020. Although the figure is unreadable in the manuscript file one should provide a list of the most frequent word for each of the years, and also say something about how this changes over time and how this should be interpreted.*

- Thank you for this suggestion. The manuscript has been amended to clarify that the most prevalent terms listed (“views,” “news,” “health,” and “love”) were the most prevalent terms for every year and major keywords in user descriptions did not vary over time. Because we found that a large proportion of users each year were newly engaged in discussions around FGM/C (described in Table 1), these findings may suggest that users with similar views, backgrounds, and intentions may be drawn to Twitter as a platform for discussing these topics. This interpretation has been added to the first paragraph of the discussion section. A full list of the most frequent words in tweet text and most frequent words in the user descriptions, as well as their prevalence for each year, have been added to the submission as supplementary tables 2 and 3.

*Likewise, on p. 6 the authors state that 34 top words in the text were distinguished. Again I would like to know what these words were and how their use changes over time.*

- Thank you for your question. We added text to the ‘Characterizing FGM/C conversations over time’ subsection of the ‘Materials and methods’ section to clarify this point- “In total, 34 words were included, as this was the number of words in the top 15 word lists after deduplicating those that repeated over years.” We also added text to the results section to identify the top terms in 2020 (as described in Figure 4), identify other common terms found in the entirety of the dataset, and clarify that term prevalence did not change significantly over time. A full list of the most frequent words in tweet text and most frequent words in the user descriptions, as well as their prevalence for each year, have been added to the submission as supplementary tables 2 and 3.

*p.6: Explain how 10 topics were selected after ' exploratory analysis' .*

- Thank you for the comment. We added the following sentence to the ‘Characterizing FGM/C conversations over time’ subsection of the ‘Materials and method’ section: “After exploratory analysis, our team decided that ten topics balanced maximizing identifiable constructs in the data and minimizing redundancy across topics.

*I do have my doubts limiting the analysis to English language tweets. It shouldn't come as a surprise that most tweets come from the US and the UK and some other large English-language countries. However, these usually are not the countries where FGM is most prevalence. In both the US and the UK the practice is banned and rare, while although both Nigeria and Kenya do know FGM the prevalence there is only 27%. Therefore, the countries where the practice is most common are excluded from the analysis because of the choice to include only tweets in English. I also wonder what the relevance of the US and UK tweets is for the struggle against FGM. This also introduces a serious bias in the data.*

- We agree that this is a limitation and note this in the ‘Discussion’. Although countries like Nigeria and Kenya have other national languages in addition to English, data shows that English is the most widely used language on Africa Twitter. Future studies on this topic will consider other languages spoken in countries with high FGM prevalence.
  - https://portland-communications.com/publications/how-africa-tweets-2016/

*On p.8 the authors report the number of users over the various year. A substantial variation is observed with especially in 2016 a huge drop in the number of users. I would like to read some tentative explanation for these fluctuations.*

- We thank the reviewer for making this observation. Unfortunately, we do not have enough information on why the number of users changed from year to year. Any explanations without information from Twitter would be speculation.

*I am surprised by the observation that the description terms do not vary substantially over time, while I would expect that terms like ' Trump' and ' MAGA' to be still relatively rare in 2015.*

- Thank you for this observation. Donald Trump formally announced his candidacy in June 2015 to substantial fanfare. Trump also registered “Make America Great Again” as a service mark with the US Patent and Trademark Office in 2015. Given Trump’s rapid rise to power and this timeline, it seems reasonable that Trump and MAGA were showing up as common phrases in user descriptions on Twitter.
  - <https://tsdr.uspto.gov/#caseNumber=85783371&caseType=SERIAL_NO&searchType=statusSearch>
  - <https://www.washingtonpost.com/politics/how-donald-trump-came-up-with-make-america-great-again/2017/01/17/fb6acf5e-dbf7-11e6-ad42-f3375f271c9c_story.html>

*What do the shifts in topics described in Table 2 mean. Please provide some tentative interpretation.*

- Thank you for your comment. On page 15 of the ‘Discussion’ section, we provide additional tentative interpretation of the shifts in topics. “Across the time period, we observe a shift from raising awareness about FGM/C (education, discussion of cultural aspects) to more explicit calls to an end of the practice. In the later portion of the study period, we see more movement-based language arise, tying FGM/C to feminism and struggles for human rights.”

**Comments from Reviewer 3:**

*The manuscript describes tweets about female genital mutilation/cutting in English from the years 2015-2020, using machine learning methods. The aim is to characterize the substance of conversations about FGM/C during these six years on Twitter and assess changes over time. The topic of public discourse of FGM/C is an interesting topic that would be of interest to many readers and I applaud the researchers for initiating this research, although I questions whether PLOS Global Public Health is the most appropriate journal. As a reviewer asked to critically examine the manuscript, I have concerns about the authors' presentation of the study, the rigor of the research methods, and hence the validity of the findings. I offer some comments below, which may serve the authors in their future work.*

*Clearly, I find that the methods are the most worrisome part of the study presented. First, the aim of the study is expressed in vague terms (page 5: “to characterize the substance of conversations about FGM/C on Twitter between 2015-2020 and assess any changes over time”).*

- Thank you for this feedback. We have edited this paragraph to clarify why we focused on the aspects of the FGM/C Twitter conversation that we did. Here is the amended paragraph: “In this paper, we use social media data to assess public perception of FGM/C on Twitter between 2015 and 2020. First, we characterize general discussions of FGM/C on English-speaking Twitter. Next, we analyze trends in discussion of FGM/C discussions on Twitter over the study period. Lastly, we characterize the meta-data of the discussion- what terms are people using to describe FGM/C, who is engaging in the conversation, and what influences engagement.”

*It is not stated how the tweets were recruited/extracted/retrieved from Twitter. It appears that the unit of analysis was a tweet addressing FGM/C (indicated by e.g. the title and the discussion stating “Tweets about FGM/C were identified based on the presence of certain terms” page 15), but it could also be Twitter user (who tweeted about FGM/C). In any case, the researchers do not state what the inclusion criteria were.*

- Thank you for your comment. We’ve added an additional ‘Data extraction’ subsection to ‘Materials and methods’ section: “We extracted tweets via Twitter’s Application Programming Interface (API), which provides a random 1% sample of all public tweets containing specific keywords. Tweets in our corpus contained at least one of the following terms: “FGM,” female genital mutilation,” “female genitalia mutilation,” “female circumcision,” “female genital alteration,” “female genitalia alteration,” “female genital cutting,” “female genitalia cutting,” “female genital excision,” and “female genitalia excision.” The research was conducted in line with Twitter API’s requirements for academic research [22]. We restricted our tweets to tweets in English. ”

*They need to explain each variable and how it was defined. On page 6 they explain that they evaluated the use of FGM/C terminology by determining the frequencies of the terms, but clearly, this must be closely link to their inclusion criteria leading to a tweet (or user) being included in the first place so this must be addressed.*

- Thank you for this suggestion. In addition to the language that we added to the subsection on data extraction, we also added this sentence to the subsection on characterizing FGM/C over time on page 10. “We explored the frequency of words used to describe FGM/C by identifying the words that we used to extract tweets.”

*On page 7 they state that tweet topics were synthesized, but they do not explain how. It appears that the machine learning methods that the researchers used were two classifiers, and they were only used to classify user engagement, but this is not clear and the two classifiers are not described.*

- Thank you for the comment. We added the following sentence to the ‘Characterizing FGM/C conversations over time’ subsection of the ‘Materials and method’ section: “After exploratory analysis, our team decided that ten topics balanced maximizing identifiable constructs in the data and minimizing redundancy across topics.” We used two classifiers, random forest and support vector machine. Our methods are described in the Understanding user engagement subsection of the methods section. “We analyzed tweet and user characteristics associated with greater user engagement using two classifiers – support vector machine (SVM) and random forest models, which have been used in similar studies [26,27]. We fit models first on all of the years in the data. We then fit models on each year's subset, using the number of retweets to represent user engagement. Number of retweets was classified into two categories: high retweets were defined as the year’s 90th retweet percentile or above, and low retweets were a random matched sample of tweets with zero retweets. We chose retweet count as the representation of user engagement because it had little missingness compared to like count, favorite count, reply count, and quote count. We split all user engagement models into train and test sets using a 70/30 split ratio.

We optimized the random forest model with varying numbers of trees and inclusion of predictors. Account level variables in the model included number of accounts followed, number of accounts following, number of tweets tweeted, and account verification. Tweet level variables included tweeting on a high volume date (defined as a top 11 date for the dataset), tweet length, and inclusion of key FGM/C terminology in the tweet (“alteration,” “circumcision,” “cutting,” “excision,” “mutilation,” “fgm”). We compared accuracy between the SVM and random forest models and ran separate models for each of the six study years.”

We then found that the random forest predicted our outcome of interest more accurately than the support vector machine model, as is described in the results section. As such, we presented only the results from the random forest model.

*On page 12, the researchers state that random forest model was used to predict engagement and they should state what the predictors in the model were and how engagement was defined.*

- Thank you for this suggestion. The specifics of the random forest and support vector machine models are included in the ‘Understanding user engagement’ subsection of the ‘Materials and methods’ section on page 8. We added a line that includes the set of variables included in the random forest model in the User engagement subsection of the Results on page 12.

*In sum, my main concern is the careless (unscientific) description of the research conducted. In the results section, I suggest the researchers follow standard reporting and start by presenting the sample. The “full dataset” is referred to on page 12 (line 239) and it is unclear if the full dataset is the 338,845 unique users they refer to on page 8 or the “large sample size … With over a million original tweets” (on page 15).*

- Thank you for bringing this to our attention. We included a more explicit sample size description in the user characteristics subsection of the results section. It’s included here: “The final dataset contained 1,301,203 tweets about FGM/C. Of these tweets, 1,004,937 were original tweets and 296,266 were retweets. During the study period from 2015 to 2020, 338,845 unique users tweeted about FGM/C.”

*They explain that the number of verified Twitter users were stable, but what does it mean in this study that a user is verified? If it means something along the lines of having the blue checkmark (to indicate active, notable, and authentic accounts that Twitter verified based on certain requirements) then it must be stated and the discussion needs to address that >90% of users in this study were NOT verified.*

- Thank you for this suggestion. We clarified this further in the ‘User characteristics’ subsection of the ‘Materials and methods’ section by stating: “We also looked at the number of users with verified accounts, that is the number of accounts that are designated by Twitter as ‘active, notable, and authentic accounts of public interest.’”

*Related, in the introduction the researchers state that “social media are uniquely positioned to track public conversations over time, particularly for sensitive topics” but what is the evidence that we can trust social media posts (tweets in this care) about a topic are expressions of people’s true sentiments?*

- Thank you for this suggestion. There is the potential for people to not portray their true sentiments on social media. This is akin to, but not exactly like, social desirability bias that appears in surveys, interviews, and focus groups. One of the strengths of social media data is that the participant is not engaging with a researcher and therefore is not attempting to answer in the way that the researcher wants to hear. However, like all other data sources about people's sentiments, social media data cannot guarantee that the participant is portraying their true sentiment. This benefit of social media data has been described in prior research.
  - <https://onlinelibrary.wiley.com/doi/full/10.1111/ppe.12622>

*In the results, the researchers should re-consider analysing based on country, given that only a third of the users (or tweets) could be linked to a country (i.e. 70% of the sample missing).*

- Thank you for your comment. We recognize that this exact text matching method leads to missingness in the data and opportunity for bias if there are user differences based on whether locations are listed, and whether these locations are reported clearly and accurately. However, because a small proportion of Tweets are geocoded (<https://www.ncbi.nlm.nih.gov/pmc/articles/PMC4636345/>), self-reported locations listed on user profiles are often used to infer user location (<https://arxiv.org/abs/2003.12309>). We believe that the proportion of user locations identified in this dataset is consistent with that identified in other studies using similar text-matching approaches (<https://link.springer.com/article/10.1007/s10707-017-0296-z>).

*Without a description of the inclusion criteria it is impossible to judge the results regarding the terms used to refer to FGM/C, but presumably to be included in the dataset certain terms used for FGM/C had to be used. It is a stretch to claim that a term used 2.2% of the time is a “commonly used” term (page 19).*

- Thank you for your comment. In response, we added the following sentence to the ‘Characterizing FGM/C conversations over time’ section of the results: “We explored the frequency of words used to describe FGM/C by identifying the words that we used to extract tweets.” We also edited this sentence in the same paragraph on page 11: “Compared In addition to female genital mutilation or FGM, cutting (4.3%), female circumcision (2.2%), excision (0.1%), and alteration (<0.1%) were less commonly used terms for this practice.”

*In table 2 the topics listed in column 1 are presumably labelled by the authors and I suggest they re-consider the labels. Judged by the tweet examples in the table, it is hard to see the difference between some of the topics, e.g. the topic ‘cultural aspects’ and ‘activism. ‘Evils of the other’ could more appropriately be labelled violence against women. It is very hard to believe that the topics of ‘medical risks’ and ‘cultural aspects’ were not topics at all in four and three years, respectively. Findings that the highest volume of FGM/C related tweets was on February 6 is obvious.*

- Thank you for your suggestion. Our tweet examples are composites of all of the quotes in the identified topic area. We edited the quote on cultural aspects to clarify that almost all quotes in this category included the idea that FGM/C was cultural. “FGM is a cultural practice- not a religious one. Culture can change.” The activism category included appeals to end FGM/C and were more likely to include calls to action. We renamed the category “Evils of the Other” to “Anti-immigrant Sentiments” to be more precise. We also edited the corresponding section in the discussion on page 15. “Another significant portion of the conversation used FGM/C as a rhetorical tool to associate FGM/C with Islam. A new topic which arose in 2017 with anti-immigrant sentiments. It connected FGM/C to other practices such as child marriage, acid attacks, female infanticide, and gang rape. These tweets referenced that these practices were not part of British or American culture and made out countries where FGM/C is common to be backwards.”

*The discussion should include less repetition of the results and more what the results mean, what are their implications, without being too speculative. The researchers should be careful drawing conclusions. First, at all, because as they state themselves “Twitter users are not representative of the global public” and second, about users’ country of residence, given they only included English language tweets. Clearly, when the absolute number of Twitter users increase, one can expect to observe also an increase in tweets addressing FGM/C. On page 14 (lines 275-276) it is incorrect to state that feminism and human rights were among the persistent topics – they are at the bottom of the table (table 2) and only identified two of the six years. Other topics are identified all or most years.*

- Thank you for your comments. Part of why we included the descriptive plots that display the number of tweets per year is for the reason that you laid out. We do not see a monotonic increase in the number of tweets about FGM/C, even though we expect the number of Twitter users to be growing over this time period. We amended the language in our discussion on page 15 to reflect that feminism and human rights are not as persistent as the other topics. “Activism and education were persistent topics that arose in the data across years. In the later portion of the study period, we see more explicit movement-based language arise, tying FGM/C to feminism and struggles for human rights.”

*In the discussion, they need to take into account the change in number of Twitter users/accounts during the years analysed.*

- Thank you for your suggestion. Unfortunately, we do not have access to the change in the number of Twitter users/accounts during the years analyzed. We included the descriptive results regarding the number of tweets and users each year to demonstrate that conversations about FGM/C are not increasing monotonically as (assumedly) number of Twitter users are.

*The authors give five implications of their findings, but they do not seem substantive. Additionally, the first appears tautological (linked to inclusion of tweets), the third is dubious because only 7% of users were verified, the fourth is redundant (those of us who have researched and been engaged with the topic of FGM/C know that FGM/C is politicized) and the fifth is not linked to any finding in this study and is normative.*

- We thank the reviewer for sharing their expertise. Our goal is to evaluate what social media - a crowdsourced pool of information from private individuals and verified entities - reflects back to us about this topic. Framing effective policy requires us to understand how the global community views this topic. We have made the changes to clarify that these are observations and not implications. Specifically, we rewrote this paragraph as follows: “These findings reveal known and novel insights about FGM/C-related public health communication and practice on Twitter. First, on English- speaking Twitter, the term FGM was most frequently used to describe the practice. Second, endfgm was the single most common word identified in our dataset. Use of this hashtag connected public health communication with the greater FGM/C advocacy conversation emphasized supporting survivors and uplifting their voices. Third, given that user verification was the best predictor of engagement, verified accounts had a particularly large role to play in engaging the public in education about FGM/C. In addition, these results suggest that the conversation about FGM/C is increasingly politicized in English- speaking contexts, particularly in the UK and US, as seen by the strong conservative voice in the conversation; a fact well-known by researchers who study FGM/C [36].”

*A few other, minor concerns are: It is not clear what they mean when they state that: the findings can be used to “leverage social media” (page 5), that the analysis is the “first of its scale” (page 5, what do they mean by ‘scale’ in this context?), “we evaluated changes in the most frequently identified countries across years” (page 6, what do they mean by changes in countries?), “phenomena that are considered ‘of the other’ in the West” (page 14). There are also several grammatical and spelling mistakes that should be corrected.*

- Thank you for your thorough reading. We have removed the sentence that states that the analysis is the first of its scale. We clarified the justification of the study by adding: “Our findings can add to the current literature about public perception about FGM/C. Further, our work can be used by medical professionals, public health practitioners, governmental agencies, and advocacy organizations seeking to educate the public about FGM/C” in the introduction on page 5. We clarified the language in most frequently identified countries to be “Using extracted country names, we calculated the proportion of total users each year who identified each country in their user location, and evaluated if the countries people are most frequently tweeting from changed across years.” As indicated above, we changed the language of “phenomena of the other” to anti-immigrant sentiment. In this instance, we changed the language to “FGM/C sentiment that connects the practice closely with other violences against women that are relatively uncommon in the US and UK may further stigmatize people who have undergone FGM/C by painting “narratives of victimhood” [35].”
